# Self-determined motivation towards physical activity in adolescents treated for obesity: an observational study

**DOI:** 10.1186/1479-5868-8-97

**Published:** 2011-09-19

**Authors:** Maïté Verloigne, Ilse De Bourdeaudhuij, Ann Tanghe, Eva D'Hondt, Lotte Theuwis, Maarten Vansteenkiste, Benedicte Deforche

**Affiliations:** 1 Department of Movement and Sport Sciences, Ghent University, Ghent, Belgium; 2 Zeepreventorium, De Haan, Belgium; 3 Department of Psychology, Ghent University, Ghent, Belgium; 4 Department of Human Biometrics and Biomechanics, Vrije Universiteit Brussels, Brussels, Belgium

## Abstract

**Background:**

Within the Self-Determination Theory (SDT) framework, the first major study aim was to investigate the SDT tenets in an obese adolescent population by examining the factor structure of the Behavioural Regulation in Exercise Questionnaire-2 (BREQ-2) and by investigating associations between physical activity (PA) and motivation in obese adolescents. The second aim was to study differences in motivation according to adolescents' educational level, since lower educated obese adolescent are a sub-risk group for lower PA levels among the obese adolescents. The third aim was to investigate whether attending a residential obesity treatment program could lead to an increase in autonomous motivation towards PA and to see if the treatment effect on motivation was different in low versus high educated youth.

**Methods:**

For the first study aim, the sample comprised 177 obese adolescents at the start of a 10-month multidisciplinary residential obesity treatment program (BMI = 35.9 ± 6.0 kg/m^2^, 15.1 ± 1.5 years, 62% girls). A subsample of 65 adolescents (stratified by educational level) were divided into low (n = 34) versus high educated (n = 31) as part of the second and third study aim. Motivation was assessed using the BREQ-2 and PA using the Flemish Physical Activity Questionnaire.

**Results:**

Exploratory factor analysis showed sufficient validations with the original factor for 17 out of 19 BREQ-2 items. Significant positive correlations were found between PA and the composite score of relative autonomy (r = 0.31, p < 0.001), introjected (r = 0.23, p < 0.01), identified (r = 0.31, p < 0.001) and intrinsic regulation (r = 0.38, p < 0.001). Higher educated adolescents scored higher on the composite score of relative autonomy, introjected, identified and intrinsic regulation at the start of treatment (F = 3.68, p < 0.001). The composite score of relative autonomy, external, identified and intrinsic regulation significantly increased during treatment for all adolescents (F = 6.65, p < 0.001). Introjected regulation significantly increased for lower educated adolescents (F = 25.57, p < 0.001).

**Conclusions:**

The BREQ-2 can be used in an obese adolescent population. Higher levels of autonomous motivation towards PA were related to higher PA levels. Adolescents had increases in both autonomous and controlled forms of motivation during treatment. Special attention for lower educated adolescents during treatment is needed, as they have a lower autonomous motivation at the start of treatment and an increase in introjected regulation during treatment.

## Background

Overweight and obesity in adolescence are associated with several adolescence and further life course physical and psychological problems [1,2]. Adolescents who already contend with overweight or obesity, are consequently impelled to follow a treatment program [3]. Physical activity (PA) is one of the key components in obesity treatment and one of the best predictors of long-term maintenance of weight loss [4]. To promote PA as an obesity treatment strategy in adolescents, a better understanding of factors that influence participation in PA in obese adolescents is important.

The Self-Determination Theory (SDT) provides insight into reasons why people adopt and maintain certain health behaviours [5,6] and has been used to understand exercise and PA participation [7]. According to the SDT, the regulation towards PA can be amotivated, extrinsically motivated or intrinsically motivated. Amotivation is a state characterized by a lack of intention to engage in the activity [8]. Extrinsic motivation implies that a person engages in the behaviour to achieve outcomes that are separable from the behaviour itself. Within extrinsic motivation there is a continuum of behavioural regulations, reflecting the degree of autonomy or self-integration. *External regulation *involves being physically active to satisfy an external requirement (e.g., rewards, sanctions, expectations). *Introjected regulation *involves motivation towards PA in order to avoid negative feelings or to enhance one's ego. Both external and introjected regulation represent controlled types of motivation as individuals will likely feel pressured to perform the behaviour [5,6]. For *identified regulation *on the contrary, the behaviour is performed more willingly even though the activity is not enjoyable. A person can participate in PA, because the behavioural outcomes are personally important, for example to improve physical fitness. The most self-determined form of the extrinsic motivation continuum is integrated regulation. The identification of the behaviour has been made consistent with the person's other values and needs. For example, some individuals might view PA as an important component of a healthy lifestyle. Although these types of extrinsic motivation attain a separable outcome than the activity itself, identified and integrated regulation involve personal endorsement of the reason to engage in the activity and, as a result, are more likely to be accompanied with feelings of choice and psychological freedom [8]. Finally, intrinsic motivation represents the most self-determined type of motivation and refers to engaging in the activity for its own sake. An intrinsically motivated person considers the PA inherently enjoyable, interesting and challenging [5,6].

External regulation and introjected regulation are typically viewed as controlling types of behavioural regulation, whereas identified and integrated regulation and intrinsic motivation represent autonomous types of behavioural regulation [8]. Research has shown that these autonomous types of behavioural regulation are associated with greater continuous PA participation [9-15]. Markland and Ingledew [16] for example found that introjected, identified and intrinsic regulation were positively related to exercise behaviour in adolescents, whereas amotivation was negatively related to their exercise behaviour. Consequently, it could be important to enhance more autonomous types of motivation to increase the continuous participation in PA. According to SDT, autonomous types of motivation stem from environments that support three psychological needs, that is the need for autonomy (i.e., experiencing a sense of psychological freedom when engaging in an activity), competence (i.e., feeling effective to attain desired outcomes) and relatedness (i.e., being socially connected). To increase the extent of autonomous motivation, it is recommended to create an environment which supports these psychological needs [8]. Increasing autonomous motivation towards PA by focusing on the three psychological needs might also be a useful strategy to increase PA levels in obese youth.

However, if we want to use the principles of SDT in obese adolescents, we have to investigate first if the association between autonomous motivation and PA is present in this specific population as the application of SDT has not been investigated yet in obese youth. In addition, the questionnaire commonly used to measure the different motivational subtypes, that is the Behavioural Regulation in Exercise Questionnaire-2 or BREQ-2 [17], has never been used in obese adolescents. Therefore, it is necessary and instructive to examine the factorial validity and predictive validity of the BREQ-2 in this specific population.

Moreover, as following a treatment program is often necessary to tackle one's obesity problem, it is interesting to examine which impact a residential treatment program has on the different types of motivation towards PA. A residential treatment program is often preferable to ambulant treatment in case of severe obesity. The permanent support from a professional team allows for dramatic weight loss [18,19]. However, to the extent such professional teams put pressure on obese individuals to engage in PA and to lose weight, the treatment team may hamper autonomous types of motivation according to the SDT. A lack of autonomous motivation may be related to relapse to unhealthy behaviours after treatment [20], which should be avoided in the interest of weight management. Studies in adults also showed that the increase in autonomous motivation towards PA is one of the strongest predictors of long-term weight loss [21,22]. However, a residential treatment does not necessarily need to be experienced as controlling. A residential treatment program could also attempt to foster the three psychological needs (autonomy, competence, relatedness). Conversely, this would imply an increase in autonomous motivation. Consequently, former findings show evidence for the importance of examining the change in different types of motivation during residential treatment in obese adolescents.

Finally, lower educated adolescents might be considered as a vulnerable subgroup within the obese adolescents, since the prevalence of overweight is higher in low educational level groups and since they are an at risk population for lower PA levels [23,24]. The lower activity levels of low educated youth might be partly explained by lower degree of autonomous motivation. However, no studies have ever compared the degree of the different types of motivation for PA in low versus high educated youth. Additionally, it might be interesting to investigate whether residential obesity treatment has a different effect on different types of motivation for PA according to educational level of the patients.

The current study has three major aims. A first aim is to investigate the applicability of the BREQ-2 among obese adolescents by investigating its factor structure. Hence, we will also investigate if PA levels of obese adolescents are related to the different motivation types. A second aim is to examine differences in the different motivation types in low versus high educated youth. The final aim is to investigate how motivation changes during a residential obesity treatment program and if the treatment effect on motivation is different in low versus high educated youth.

## Methods

### Procedure

All patients (> 12 years old) entering the residential weight reduction treatment between January 2007 and July 2008 completed the Behavioural Regulation in Exercise Questionnaire (BREQ-2) and the Flemish Physical Activity Questionnaire (FPAQ) under supervision of the physiotherapist of the centre. In total, 177 adolescents completed the questionnaires in the scope of our first study aim. Body weight and height were measured by the medical doctor of the centre. Since the adolescents were overloaded with physical and medical tests, psychological questionnaires and anthropometric measurements at the end of the residential treatment, it was impossible to have all adolescents fill in the BREQ-2 again. Therefore, a random subsample of 65 adolescents (stratified by educational level) completed the BREQ-2 again as part of the second and third study aim. Adolescents' PA level was not assessed at the end of the treatment, since every adolescent had followed the same activity program for the previous 10 months. The study protocol was approved by the ethical committee of the Ghent University Hospital. Informed consent was obtained from the treatment centre, parents and youngsters.

### Participants

Table 1 presents the characteristics of the whole sample and the subsample. The participants of this subsample did not significantly differ from the whole sample in weight, BMI (z-value), gender and nationality, although they were a bit older (p = 0.063) and a bit taller (p = 0.02). Participants of the subsample were classified in a lower educational level group (vocational, technical, art and special education; n = 34) and a higher educational level group (general secondary education; n = 31). Participants attended a 10-month inpatient obesity treatment program in a local centre (Zeepreventorium, De Haan, Belgium). This multi-component program consisted of moderate dietary restriction (1600-1800 kcal/day), regular PA and cognitive behavioural techniques. The exercise program included 4 hours per week of exercise with a physiotherapist, 2 hours of physical education per week at school and 2 hours of supervised games and lifestyle activities per day before and after school. Physical therapists and educators tried to fulfill the three psychological needs (autonomy, competence and relatedness) by giving them the choice between activities, by working with small, realistic objectives in order to experience success and by creating a strong bond between them and the adolescents. The integration into a peer group with similar problems might enhance the basic need of relatedness too. Further, adolescents received group and individual psychological support and medical supervision (without medication). Participants attended school in the residential setting (i.e., special education for chronically ill children) and were allowed to return home every weekend, except one per month, and during half of each school holiday period. Parental involvement was consequently limited during the program. This treatment program has previously been shown effective in decreasing overweight and increasing fitness [18,19]. Criteria for entry to the treatment program included a minimum of 40% overweight, no endogenous cause of obesity and a normal intelligence quotient (IQ > 70).

**Table 1:** Characteristics of the total sample and subsample

	Total sample (n = 177)	Subsample (n = 65)
Age	15.1 ± 1.5 y	15.5 ± 1.4 y
Sex	62% girls	59% girls
Height	166.5 ± 8.3 cm	169.0 ± 9.2 cm
Weight	99.9 ± 19.8 kg	102.7 ± 19.5 kg
BMI	35.9 ± 6.0 kg/m^2^	35.9 ± 5.7 kg/m^2^
z-BMI	2.65 ± 0.4	2.62 ± 0.4
Nationality	97% Belgian nationality	95% Belgian nationality

### Measures

#### Anthropometrical measures

Body weight was measured to the nearest 0.1 kg with a digital balance scale (SECA, maximum 200 kg, Hamburg, Germany) with the adolescent wearing light weight clothing and no shoes. Height was measured to the nearest 0.1 cm with a stadiometer (Holtain LTD, Crymmych, Pembs, UK). The BMI in kilogram per square meter (kg/m^2^) was calculated on the basis of height (m) and weight (kg) measures. BMI z-scores were calculated on the basis of the Flemish reference data using the LMS method [25].

#### Level of PA

PA level was determined using the Flemish PA Questionnaire, which has been previously validated [26]. To assess active transportation, minutes spent in active transportation to school and in leisure time were added up. Sport participation was created by adding up minutes spent in sports at school and minutes spent in physical activities during leisure time. Total PA was assessed by adding up minutes spent in active transportation and time spent in sports.

#### Behavioural Regulation in Exercise Questionnaire

The Behavioural Regulation in Exercise Questionnaire-2 (BREQ-2) has been used to measure the motivation towards exercise and showed sufficient validity in adults [17]. PA recommendations refer to all physical activities and not to exercise in particular which is only one part of PA. Therefore, we preferred to replace 'exercise' by 'PA' in the questionnaire. The BREQ-2 has been translated to Dutch by means of the translation-back translation method. The Dutch BREQ-2 has already been used in previous research [27]. The questionnaire comprises 19 items relating to five motivation types from the SDT, that is amotivation (e.g. "I don't see the point in being physically active"), external regulation (e.g. "I am physically active because other people say I should"), introjected regulation (e.g. "I feel guilty when I'm not physically active"), identified regulation (e.g. "I'm physically active because I value the benefits of physical activity") and intrinsic motivation (e.g. "I'm physically active because it's fun). Integrated regulation is not measured by the BREQ-2, because integrated regulation was not empirically distinguishable from identified and intrinsic regulation [28]. Each item is measured on a five-point Likert-scale, from 0 ('Not true for me') to 4 ('Very true to me'). The mean of the 5 subscales is usually calculated on a five-point scale to form an idea of the extent of each motivation type separately. The Relative Autonomy Index (RAI) can be used to gain insight in the degree of relative autonomy given that the five motivation types are located on the self-determination continuum. The RAI is calculated by weighting each subscale and summing the weighted scores: (amotivation multiplied by -3) + (external regulation multiplied by -2) + (introjected regulation multiplied by -1) + (identified regulation multiplied by 2) + (intrinsic regulation multiplied by 3). The minimum score for the RAI is -24 and the maximum score is +20. Higher positive scores for the RAI indicate more autonomous motivation whereas lower negative scores indicate less autonomous motivation. In brief, the RAI is the composite score of relative autonomy.

### Statistical analyses

SPSS 15.0 was used for data analysis (SPSS Inc, Chicago, IL). An exploratory factor analysis of principal components with varimax rotation was executed to investigate the BREQ-2 factor structure. To determine the number of factors to retain, SPSS used the eigenvalue > 1 rule [29]. An item with a factor loading higher than 0.40 on a factor was considered to load sufficiently high on the relevant factor. Cronbach's alpha's were calculated to determine internal consistency of the items of the retained factors. Correlations between the motivation types mutually and between motivation (the composite score of relative autonomy and the five motivation types) and PA (total PA, sport participation and active transportation) were analyzed using Pearson correlations. A multivariate analysis of variance (MANOVA) was executed to investigate the differences in the composite score of relative autonomy towards PA and the five motivation types among the high and low educational level group. To study the change in the composite score of relative autonomy and the motivation types over time, Repeated Measures MANOVA were executed with educational level of the adolescent included as a between-subjects factor. Statistical significance level was set at p < 0.05 for all analyses. A p-value ≥ 0.05, but < 0.1 was considered borderline significant.

## Results

### Investigating the tenets of SDT among obese adolescents

#### Exploratory factor analysis of the BREQ-2

Table 2 presents the results of the exploratory factor analysis. Based on the eigenvalues, five factors were retained with an eigenvalue above 1 with a total variance explained of 63.19%. The sixth factor had an eigenvalue of 0.92. Although the majority of the items loaded on its intended theoretical factor, a number of exceptions can be noted. Item 2 (i.e., 'I feel guilty when I don't do physical activities') showed low saturation with its original factor introjected regulation, but loaded on the retained factor identified regulation. Item 17 (i.e., 'I get restless if I don't do physical activities regularly') showed low saturation with its original factor identified regulation but loaded significantly on the retained introjected regulation factor. Finally, four items (i.e., 3, 13, 14, and 19) showed cross-loadings of more than 0.40 with other factors. All Cronbach α-values ranged between 0.64 and 0.86 for the retained factors in the factor solution. Cronbach α was also calculated for the original subscales, as suggested by the BREQ-2. Internal consistency reliability was moderate to high for the 5 subscales: Cronbach α-values ranged between 0.61 and 0.88. The original structure of the BREQ-2 was used for further analyses.

**Table 2:** Exploratory factor analysis on the Behavioural Regulation in Exercise Questionnaire-2

Factor	1	2	3	4	5	h^2^
*1. Intrinsic regulation*						
4. I do physical activities because it's fun	0.78	-	-	-	-	0.68
10. I enjoy my physical activity sessions	0.8	-	-	-	-	0.77
15. I find physical activity a pleasurable activity	0.79	-	-	-	-	0.73
18. I get pleasure and satisfaction from participating in physical activity	0.78	-	-	-	-	0.73
*2. Amotivation*						
5. I don't see why I should have to do physical activities	-	0.72	-	-	-	0.64
9. I can't see why I should bother doing physical activities	-	0.83	-	-	-	0.72
12. I don't see the point in doing physical activities	-	0.81	-	-	-	0.7
19. I think doing physical activities is a waste of time	*-0.52*	0.43	-	-	-	0.56
*3. External regulation*						
1. I do physical activities because other people say I should	-	-	0.76	-	-	0.58
6. I do physical activities because my friends/family/partner say I should	-	-	0.68	-	-	0.49
11. I do physical activities because others will not be pleased with me if I don't	-	-	0.71	-	-	0.56
16. I feel under pressure from my friends/family to do physical activities	-	-	0.52	-	-	0.55
*4. Identified regulation*						
3. I value the benefits of physical activity	*0.48*	-	-	0.66	-	0.68
8. It's important to me to do physical activities regularly	-	-	-	0.77	-	0.66
14. I think it is important to make the effort to do physical activities regularly	-	-	-	0.54	*0.48*	0.61
17. I get restless if I don't do physical activities regularly	-	-	-	0.06	*0.77*	0.62
*5. Introjected regulation*						
2. I feel guilty when I don't do physical activities	-	-	-	*0.6*	0.13	0.49
7. I feel ashamed when I miss my physical activities	-	-	-	-	0.53	0.73
13. I feel like a failure when I haven't done physical activities in a while	*0.46*	-	-	-	0.69	0.51
Eigenvalue	5.56	2.66	1.46	1.29	1.05	-
Factor variance	29.26	13.89	7.66	6.79	5.51	-
Total variance	29.26	43.24	50.9	57.68	63.19	-
Reliability 1	0.86	0.76	0.64	0.68	0.64	-
Reliability 2	0.88	0.77	0.64	0.61	0.65	-

Reliability 1 = reliability from the obtained factor structure; Reliability 2 = reliability from the original subscales

#### Correlations between the motivation types

Table 3 presents the bivariate Pearson's correlations between the five motivation types mutually. All motivation types were significantly related to each other (all at p < 0.05), except for external and identified regulation and for external and intrinsic regulation. The correlations among the subscales conformed to a simplex-like pattern with stronger positive correlations between subscales more adjacent on the self-determination continuum (e.g. identified and intrinsic regulation: r = 0.53) and stronger negative correlations between subscales more distant on the continuum (e.g. amotivation and intrinsic regulation: r = - 0.48).

**Table 3:** Pearson correlations between the motivation types

n = 177	Amotivation	External regulation	Introjected regulation	Identified regulation
External regulation	0.24**	-	-	-
Introjected regulation	-0.17*	0.23**	-	-
Identified regulation	-0.31***	0.04	0.56***	-
Intrinsic regulation	-0.48***	0.03	0.44***	0.53***

* p ≤ 0.05; ** p < 0.01; *** p < 0.001

#### Correlation between motivation and PA

Table 4 presents bivariate Pearson's correlations between PA and motivation. Statistical analyses indicated significant positive correlation between total PA and the composite score of relative autonomy (RAI) (p < 0.001), introjected (p < 0.01), identified (p < 0.001) and intrinsic regulation (p < 0.001) There were no significant correlations with total PA for amotivation and external regulation. For sport participation, a significant positive correlation was found for the composite score of relative autonomy (RAI) (p < 0.001), introjected regulation (p < 0.001), identified regulation (p < 0.001) and intrinsic regulation (p < 0.001), whereas a significant negative correlation was found for amotivation (p < 0.01). Active transportation was positively associated with the composite score of relative autonomy (RAI) (p < 0.05), identified (p < 0.01) and intrinsic regulation (p < 0.01).

**Table 4:** Pearson correlations between motivation and PA

n = 177	Total PA	Sport Participation	Active transportation
Composite score of relative autonomy (RAI)	0.29***	0.33***	0.18*
Amotivation	-0.11	-0.22**	-0.02
External regulation	0.08	-0.05	0.06
Introjected regulation	0.23**	0.32***	0.11
Identified regulation	0.31***	0.29***	0.24**
Intrinsic regulation	0.38***	0.41***	0.25**

* p ≤ 0.05; ** p < 0.01; *** p < 0.001

### Investigating differences in motivation according to educational level

Multivariate analyses indicated a significant difference in the composite score of relative autonomy (RAI) and the motivation types according to educational level (F = 3.68, p < 0.01). Univariate analyses showed a significant difference in the composite score of relative autonomy (RAI) according to educational level (F = 6.30, p < 0.05), with a higher composite score of relative autonomy (RAI) in higher educated adolescents. Both groups also differed in introjected (F = 10.04, p < 0.01) and intrinsic regulation (F = 11.21, p < 0.01) to PA, with higher scores among adolescents with a higher educational level. The difference in identified regulation between both groups was of borderline statistical significance (F = 3.17, p < 0.1) with a higher score for identified regulation for higher educated adolescents. No difference was found for amotivation (F = 1.74, ns) and external regulation (F = 1.32, ns).

### Change in motivation types after a residential obesity treatment program (according to educational level)

Multivariate analyses indicated a change in the composite score of relative autonomy (RAI) and motivation types over time (F = 8.08, p < 0.001, see table 5). Univariate analyses showed a significant change over time for the composite score of relative autonomy (RAI) (F = 9.91, p < 0.01), introjected (F = 14.97, p < 0.001), identified (F = 37.86, p < 0.001) and intrinsic regulation (F = 15.40, p < 0.001). Change over time for external regulation was of borderline significance (F = 3.06, p < 0.1). Autonomous motivation, external, introjected, identified and intrinsic regulation all showed an increase over time. Amotivation did not significantly change over time (F = 2.55, ns). Analyses indicated a significant difference in the change in introjected regulation according to educational level (F = 7.26, p < 0.01, see table 5). There was a significant increase in the extent of introjected regulation for lower educated adolescents (F = 25.57, p < 0.001), whereas no significant change was found for higher educated adolescents (F = 0.65, ns). No other significant differences in the change of motivation according to educational level were found. However, mean values of the composite score of relative autonomy (RAI) and motivation types at the end of the treatment showed a high increase for lower educated adolescents. Therefore, we investigated differences in motivation at the end of the treatment according to educational level by means of MANOVA. The analysis revealed no significant difference in the composite score of relative autonomy (RAI) or motivation types at the end of the treatment according to educational level (F = 58.00, ns).

**Table 5:** Change in motivation - Repeated Measures MANOVA

n = 65	PRE low edu (mean ± SD)	PRE high edu (mean ± SD)	POST low edu (mean ± SD)	POST high edu (mean ± SD)	F-value (time*edu)	F-value (time)
Composite score of relative autonomy (RAI)¹	2.7 ± 6.3	5.7 ± 6.1	7.1 ± 7.1	7.9 ± 6.3	1.09	9.91**
Amotivation^2^	0.9 ± 0.8	0.6 ± 0.8	0.5 ± 0.7	0.6 ± 1.0	1.86	2.55
External regulation^2^	0.9 ± 0.8	1.1 ± 0.7	1.2 ± 1.1	1.2 ± 1.0	0.56	3.06(*)
Introjected regulation^2^	0.9 ± 0.8	1.5 ± 0.8	2.0 ± 0.9	1.8 ± 1.1	7.26**	14.97***
Identified regulation^2^	1.6 ± 0.9	1.9 ± 0.8	2.7 ± 0.8	2.7 ± 0.8	2.00	37.86***
Intrinsic regulation^2^	1.6 ± 1.0	2.4 ± 1.0	2.5 ± 1.1	2.8 ± 0.8	2.50	15.40***
Total PA level (min/day)	56.2 ± 33.4	74.3 ± 39.5	-	-	-	-

edu = education; ¹ [-24,+20]; ^2 ^[0,4]; (*) 0.05 ≤ p < 0.1; ** p < 0.01; *** p < 0.001

## Discussion

The first major aim of the current study was to investigate the SDT tenets in an obese adolescent population. First, we executed an exploratory factor analysis of principal components to examine the factor structure of the BREQ-2. Results revealed that two items failed to load on their intended original factor. The low loadings of item 17 (i.e., 'I get restless if I don't do physical activities regularly') with its original factor 'identified regulation' has already been found in previous studies [10,30,31]. Item 2 (i.e., 'I feel guilty when I don't do physical activities), which taps into feelings of guilt, also failed to load on its intended introjected factor. Instead, the retained introjected factor primarily yielded a reference to the avoidance of feelings of shame and failure. Because these are prominent among obese adolescents who feel ashamed of their figure and weight [32], these items seem to cluster apart from items tapping into feelings of guilt. These findings are consistent with other authors' claim that feelings of shame and guilt need to be distinguished given their different antecedents and consequences [33,34]. In general, it is notable that items that are crossing the distinction between controlled and autonomous motivation were found to yield wrong loadings or cross-loadings. Indeed, the difference between introjected and identified regulation does not represent a sharp line, but rather represents a gradual change away from inner pressures to personal convictions. Along similar lines, Mullan et al. [35] reported that introjected regulation correlated more strongly with the more self-determined identified subscale than it did with the less self-determined external subscale.

The low validation scores of item 2 and 17 and the various cross-validation scores could be due to the fact that 177 adolescents is a relatively small sample to investigate the factor structure of a questionnaire with 19 items as it is suggested to have ten participants per questionnaire item or to have at least 200 participants [36,37]. A possible strategy to deal with the low validation scores or cross-validation scores is to exclude those specific items from the subscale calculation. However, since the BREQ-2 is strongly validated in other populations [10,17,30,31] and since the BREQ-2 has been used for the first time in an obese adolescent population, it was preferred to use the current classification. Moreover, internal consistency was rather similar using factor structure suggested by exploratory factor analysis or the current classification.

The second part of the first study aim showed that the association between autonomous types of motivation and PA was present in obese adolescents. Results showed that higher levels of the composite score of relative autonomy, identified and intrinsic regulation were related to higher amounts of total PA, sport participation and active transportation. Introjected regulation was also positively related to total PA and sport participation. These results in severely obese adolescents are similar to results of previous studies in normal-weight adolescents and in normal-weight and obese adults [9-16]. Despite the positive association between introjected regulation and PA among the obese adolescents, it should be noted that introjected regulation is a more controlled form of motivation. Previous studies have shown that introjected regulation appears to be associated with PA on the short-term, but not on the long-term [38,39]. This implies the need for a persistent emphasis on the pleasure and personal benefits associated with PA to prevent a dominant internal obligation to be physically active [40]. Amotivation was negatively associated with sport participation among the obese adolescents, which is comparable to the study of Markland and Ingledew [16] in normal-weight adolescents. Overall, we can conclude that higher levels of autonomous motivation are related to higher amounts of PA in obese adolescents.

Recommendations to increase autonomous types of motivation could therefore be used in obesity treatment programs with the intention to increase PA levels of obese adolescents. According to SDT, an environment which fosters the psychological needs for autonomy, competence and relatedness is a prerequisite to increase autonomous motivation [8,41]. In practice, more autonomy can be obtained by providing choices, supporting the patients' initiatives, avoiding the use of external rewards, offering relevant information for changing behaviour and using autonomy supportive language (e.g. "may" and "could" rather than "should" and "must") [41-43]. A feeling of competence is attained when the youngsters experience success while participating in activities. Activities need to be tailored to the capabilities of the obese adolescent and sufficient instructions, practice and positive feedback are needed to obtain a sense of competence [9,41,43]. Finally, relatedness with the supervisor or therapist and the other peers is important. Supervisors and therapists need to show enjoyment, enthusiasm and interest in the obese adolescents [43-45]. Group sessions and group activities could increase the feeling of relatedness and decrease the feeling of being isolated [43]. Former recommendations should be taken into account during an obesity treatment program to enhance autonomous motivation towards PA in obese adolescents.

The second aim was to investigate differences in the composite score of relative autonomy and the motivation types in low versus high educated obese adolescents. Results revealed that lower educated youngsters had a lower score on the composite score of relative autonomy and showed less introjected, identified and intrinsic regulation at the start of the obesity treatment program. A possible explanation for the difference in motivation could be situated in the environment of the lower educated adolescents. For example, lower educated people have lower perceived competence to produce desired outcomes such as PA behaviour [46], probably because they are provided with less relevant information about how to change their behaviour. Further, lower educated adolescents mostly have restricted access to resources and sports facilities [24], thereby missing opportunities to be physically active. These findings do not contribute to the fostering of the need for autonomy and competence. The need for relatedness is less satisfied either, since lower educated adolescents get less support for being physically active from their social network [24]. In conclusion, the physical and social environment of lower educated adolescents is less likely to support the need for autonomy, competence and relatedness which could have negative consequences for the autonomous motivation towards PA. Consequently, lower educated obese adolescents could be at major risk of not being sufficiently physically active to maintain weight loss after treatment because of their lower autonomous motivation. Therefore, special attention concerning satisfaction of the need for autonomy, competence and relatedness is required for this group during the treatment in order to increase their autonomous motivation towards PA.

The third study aim investigated whether attending a residential obesity treatment program focusing on the three psychological needs could lead to an increase in autonomous motivation towards PA. Results showed that obese adolescents had a significant increase in the composite score of relative autonomy and in identified and intrinsic regulation after treatment. No change over time was found for amotivation. Evidence is provided for the effectiveness of a residential obesity treatment program, characterized by a well-structured environment with continuous supervision of a professional team, in increasing more autonomous types of motivation towards PA, provided that attention is paid to autonomy, competence and relatedness. To our knowledge, no studies previously investigated the change in autonomous motivation among obese adolescents following a residential obesity treatment program. However, similar research was conducted in obese adults following an ambulant obesity treatment program. Silva et al. [41,47] investigated the impact of a 1-year weight management intervention with 30 group sessions for obese women. The intervention was based on SDT with a special focus on increasing autonomous regulation towards exercise and weight control in an autonomy-supportive environment. Results of that study revealed a significant increase in exercise intrinsic motivation and autonomous motives to exercise at the end of the treatment. Conversely, in a study of Edmunds et al. [37], obese female adults taking part in regular exercise classes had no significant change in intrinsic motivation and even a decrease in identified regulation, possibly due to unrealistic weight loss expectations. These findings suggest that an obesity treatment program should specifically focus on satisfying the need for autonomy, competence and relatedness to increase the autonomous motivation towards PA.

Despite the positive results for the more autonomous types of motivation in the present study, it should be noted that there was a significant increase in introjected regulation and even a borderline significant increase in external regulation as well. Thus, the residential treatment program might have put pressure on the adolescents to become physically active, which has contributed to the increase in external and introjected regulation. The increases could also be partly explained by the increases in autonomous forms of motivation since these forms are interrelated. For example, introjected regulation was shown to relate positively to both identified and intrinsic regulation, which has been found by previous studies as well [10,13,28]. As a result, the adolescents of the present study did not only have an increase in autonomous forms of motivation, but also in controlled forms of motivation towards PA. Thus, adolescents' overall motivation increased. This suggests that the residential program may contain a mix of controlling and need-thwarting components and more need-supportive features, although future research may want to directly tap into the experience of the social environment. In a recent study of Haerens et al. [27], normal-weight college students with high scores on both autonomous and controlled motivation towards PA (i.e. high quantity motivation), engaged less in PA than their contemporaries with high scores on autonomous motivation and low scores on controlled motivation towards PA (i.e. high quality motivation). From this study, it can be concluded that the quality of motivation is more important than the quantity. If these findings can be generalized to obese adolescents, it is important that a residential obesity treatment program focuses primarily on increasing the autonomous forms of motivation and minimizes control to enhance PA behaviour.

Additionally, we wanted to investigate if the treatment effect on motivation was different in low versus high educated youth. Results revealed that the change in the composite score of relative autonomy and the motivation types was not significantly different for lower and higher educated youngsters, except for introjected regulation. Lower educated youngsters had a significant increase in introjected regulation during the course of the treatment, whereas no change in introjected regulation was found among the higher educated adolescents. As mentioned before, introjected regulation seems to be positively related to PA only on the short-term [38,39], which highlights again the special attention required for the lower educated adolescents during the treatment program. However, concerning the other motivation types, mean values showed that the lower educated adolescents kept pace with the higher educated adolescents after treatment. Thus, although lower educated adolescents had lower autonomous motivation at the start of the treatment, this difference in autonomous motivation according to educational level was no longer present at the end of the treatment. Consequently, the treatment program cleared away the differences in motivation between lower and higher educated adolescents in the course of the program, thereby decreasing socio-economic inequalities. Further research should investigate possible changes in autonomous motivation towards PA among lower educated adolescents when they end the treatment and return to their home environment.

There are some limitations in the present study that need to be acknowledged. A first limitation is the cross-sectional observational design through which we cannot rule out the possibility that the association between autonomous types of motivation and PA represented reverse causality and that a higher PA level could have led to more autonomous motivation towards PA. Notwithstanding the previously demonstrated reliability and validity of the measures, the use of self-report measures can be seen as a second limitation. Particularly the self-report of adolescents' PA level could involve overestimation: the completion of the FPAQ took place in the local centre under supervision of the physiotherapist which could have led to social desirable answers. However, the presence of the physiotherapist can also be considered positively, since he or she could clarify vague questions as well as check if all questions were completed. Nevertheless, it can be concluded that accelerometers or other objective motion sensors would have been more appropriate and accurate PA measurements. Moreover, using objective motion sensors would have had the advantage to detect differences in PA at the end of treatment, which was now useless to measure by means of the FPAQ because of the standard activity program for all adolescents at the treatment centre. It should also be notified that using the BREQ-2 in younger obese samples could require adjustments as regards calculations of the five motivation types according to the exploratory factor analysis, despite a similar internal consistency when using the current classification. It can be argued that a confirmatory factor analysis might be a preferred method to examine to factor structure of the BREQ-2 given its ability to test a priori theory. However, we were unable to conduct such analysis because of the relatively low study sample. Further, the present study has not investigated the cause of the increase in autonomous motivation during obesity treatment. Future research should therefore examine which specific factors mediate the increase in autonomous types of motivation during treatment (e.g. increase in psychological need satisfaction, increase in fitness, loss of body mass, etc.). A final limitation could be the relatively small sample size and the very specific population of extreme obese adolescents in a residential setting, thereby limiting the extent to which findings can be generalized to all obese adolescents. However, the specificity of the study population can also be seen as a strength, since the significant results of this study demonstrated the universality of the application of SDT. Further, to our knowledge, no other study has previously investigated the application of SDT in lower versus higher educated individuals, which can be seen as a valuable strength of this study.

## Conclusions

The previously validated BREQ-2 can be used in obese adolescents. If so desired, small adjustments can be made to the questionnaire. Moreover, a positive association was found between autonomous motivation and PA in obese adolescents who were at the start of a residential obesity treatment program. Higher levels of autonomous motivation towards PA were related to higher amounts of PA. Providing that attention is paid to the satisfaction of the need for autonomy, competence and relatedness, attending a residential obesity treatment program might increase autonomous forms of motivation towards PA during treatment. However, it should be noted that the strictly controlled environment of the residential treatment program could have engendered increases in controlled forms of motivation. Residential treatment programs are therefore advised to take these findings into consideration and to try to minimize control as far as possible. During treatment, it appeared that the lower educated adolescents kept up with the higher educated ones with regard to autonomous motivation. Nevertheless, it might be important that treatment program staff pays special attention to lower educated adolescents because of their lower scores on autonomous forms of motivation observed at the start of the treatment and their increase in introjected regulation during treatment. Silva et al. [39] have already shown that autonomously motivated overweight and obese women were able to remain physically active and maintain their weight loss after three years. Future research should now investigate whether the more autonomously motivated obese adolescents are more physically active on the long-term and are able to maintain their weight loss as well.

## Competing interests

The authors declare that they have no competing interests.

## Authors' contributions

BD and IDB conceived the study. MVe has conducted the analyses and wrote the first draft of the paper. EDH, LT, MVa, IDB and BD have significantly contributed to the final manuscript by introducing new research questions and discussion points. AT has collected the data. All authors read and approved the final manuscript.

## References

[B1] Lobstein T Baur L Uauy R IASO International Obesity Task Force Obesity in children and young people: a crisis in public health Obes Rev 2004 5 4 104 10.1111/j.1467-789X.2004.00133.x 15096099

[B2] Warschburger P The unhappy obese child Int J Obes 2005 29 S127 S129 10.1038/sj.ijo.0803097 16385764

[B3] Tsiros MD Sinn N Coates AM Howe PRC Buckley JD Treatment of adolescent overweight and obesity Eur J Pediatr 2007 167 9 16 10.1007/s00431-007-0575-z 17973118

[B4] Jeffery M Wing RR Sherwood NE Tate DF Physical activity and weight Loss: does prescribing higher physical activity goals improve outcome? Am J Clin Nutr 2003 78 184 189 10.1093/ajcn/78.4.684 14522725

[B5] Deci EL Ryan RM Intrinsic motivation and self-determination in human behaviour 1985 New York: Plenum Press

[B6] Deci EL Ryan RM The "what" and "why" of goal pursuits: Human needs and the self-determination of behavior Psychol Inquiry 2000 11 227 268 10.1207/S15327965PLI1104_01

[B7] Ryan RM Frederick CM Lepes D Rubio N Sheldon KM Intrinsic motivation and exercise adherence Int J Sport Psychol 1997 28 335 354

[B8] Ryan RM Deci EL Intrinsic and extrinsic motivations: classic definitions and new directions Contemp Educ Psychol 2000 25 54 67 10.1006/ceps.1999.1020 10620381

[B9] Gillison FB Standage M Skevington SM Relationships among adolescents' weight perceptions, exercise goals, exercise motivation, quality of life and leisure-time exercise behaviour: a self-determination theory approach Health Edu Res 2006 21 836 847 10.1093/her/cyl139 17101718

[B10] Ingledew DK Markland D The role of motives in exercise participation Psychol Health 2008 23 807 828 10.1080/08870440701405704 25160882

[B11] Mullan E Markland D Variations in self-determination across the stages of change for exercise in adults Motiv Emot 1997 21 349 362 10.1023/A:1024436423492

[B12] Standage M Sebire SJ Loney T Does exercise motivation predict engagement in objectively assessed bouts of moderate-intensity exercise?: a Self-Determination Theory perspective J Sport Exerc Psychol 2008 30 337 352 18723896 10.1123/jsep.30.4.337

[B13] Thøgerson-Ntoumani C Ntoumanis N The role of self-determined motivation in the understanding of exercise-related behaviours, cognitions and physical self-evaluations J Sports Sci 2006 24 393 404 10.1080/02640410500131670 16492603

[B14] Wilson PM Rodgers WM The relationship between exercise motives and physical self-esteem in female exercise participants: an application of the Self-Determination Theory J Appl Biobehav Res 2002 7 30 43

[B15] Wilson PM Rodgers WM The relationship between perceived autonomy support, exercise regulations and behavioral intentions in women Psychol Sport Exerc 2003 5 229 242

[B16] Markland D Ingledew DK The relationships between body mass and body image and relative autonomy for exercise among adolescent males and females Psychol Sport Exerc 2007 8 836 853 10.1016/j.psychsport.2006.11.002

[B17] Markland D Tobin V A modification to the behavioural regulation in exercise questionnaire to include an assessment of amotivation J Sport Exerc Psychol 2004 26 191 196

[B18] Braet C Tanghe A Decaluwe V Moens E Rosseel Y Inpatient treatment for children with obesity: weight loss, psychological well-being and eating behaviour J Pediatr Psychol 2004 29 519 529 10.1093/jpepsy/jsh054 15347700

[B19] Deforche B De Bourdeaudhuij I Debode P Vinaimont F Hills AP Verstraete S Bouckaert J Changes in fat mass, fat-free mass and aerobic fitness in severely obese children and adolescent following a residential treatment programme Eur J Pediatr 2003 162 616 622 10.1007/s00431-003-1247-2 12811554

[B20] Markland D Hardy L On the factorial and construct validity of the intrinsic motivation inventory: conceptual and operational concerns Res Q Exerc Sport 1997 68 20 32 9094760 10.1080/02701367.1997.10608863

[B21] Teixeira PJ Going SB Sardinha LB Lohman TG A review of psychosocial pre-treatment predictors of weight control Obes rev 2005 6 43 65 10.1111/j.1467-789X.2005.00166.x 15655038

[B22] Teixeira PJ Going SB Houtkooper LB Cussler EC Metcalfe LL Blew RM Sardinha LB Lohman TG Exercise motivation, eating, and body image variables as predictors of weight control Med Sci Sports Exerc 2006 38 179 188 10.1249/01.mss.0000180906.10445.8d 16394972

[B23] Vissers D Devoogdt N Gebruers N Mertens I Truijen S Van Gaal L Overweight in adolescents: differences per type of education. Does one size fit all? J Nutr Educ Behav 2008 40 65 71 10.1016/j.jneb.2007.06.010 18314081

[B24] Geckova A van Dijk JP Groothoff JW Post D Socio-economic differences in health risk behaviour and attitudes towards health risk behaviours among Slovak adolescents Soz Präventivmed 2002 47 233 239 10.1007/BF01326404 12415927

[B25] Roelants M Hauspie R Hoppenbrouwers K References for growth and pubertal development from birth to 21 years in Flanders, Belgium Ann Hum Biol 2009 36 680 694 10.3109/03014460903049074 19919503

[B26] Philippaerts RM Matton L Wijndaele K Balduck A-L De Bourdeaudhuij I Lefevre J Validity of a physical activity computer questionnaire in 12- to 18-year-old boys and girls Int J Sports Med 2006 27 131 136 10.1055/s-2005-837619 16475059

[B27] Haerens L Kirk D Cardon G De Bourdeaudhuij I Van Steenkiste M Motivational profiles for secondary school physical education and its relationship to the adaption of a physically active lifestyle among university students Eur Phys Educ Res 2010 16 117 139 10.1177/1356336X10381304

[B28] Brickell TA Chatzisarantis NLD Using self-determination theory to examine the motivational correlates and predictive utility of spontaneous exercise implementation intentions Psychol Sport Exerc 2006 8 758 770

[B29] Kaiser HF The application of electronic computers to factor analysis Educ Psychol Meas 1960 20 141 151 10.1177/001316446002000116

[B30] Moustaka FC Vlachopoulos SP Vazou S Kaperoni M Markland DA Initial validity evidence for the Behavioral Regulation in Exercise Questionnaire-2 among Greek exercise participants Eur J Psychol Assess 2010 26 269 276 10.1027/1015-5759/a000036

[B31] Murcia JAM Gimeno EC Camacho AM Measuring self-determination motivation in a physical fitness setting: validation of the Behavioral Regulation in Exercise Questionnaire-2 (BREQ-2) in a Spanish sample J Sports Med Phys Fitness 2007 47 366 374 17641607

[B32] Sjöber RL Nilsson KW Leppert J Obesity, shame, and depression in school-aged children: a population-based study Pediatrics 2005 116 e389 e392 10.1542/peds.2005-0170 16140683

[B33] Conradt M Dierk JM Schlumberger P Rauh E Hebebrand J Rief W Development of the Weight- and Body-Related Shame and Guilt scale (WEB-SG) in a nonclinical sample of obese individuals J Pers Assess 2007 88 317 327 10.1080/00223890701331856 17518553

[B34] Tangney JP Conceptual and methodological issues in the assessment of shame and guilt Behav Res Ther 1996 34 741 754 10.1016/0005-7967(96)00034-4 8936757

[B35] Mullan E Markland D Ingledew DK A graded conceptualisation of self-determination in the regulation of exercise behaviour: development of a measure using confirmatory factor analytic procedures Person Individ Diff 1997 23 745 752 10.1016/S0191-8869(97)00107-4

[B36] Gorsuch RL Factor analysis 1983 2 Hillsdale, NJ: Erlbaum

[B37] Jöreskog KG Sörbom D LISREL 7: User's reference guide 1989 Mooresville, IN: Scientific Software

[B38] Pelletier LG Fortier MS Vallerand RJ Brière NM Associations among perceived autonomy support, forms of self-regulation, and persistence: a prospective study Motiv Emot 2001 25 279 306 10.1023/A:1014805132406

[B39] Silva MN Markland D Carraça EV Vieira PN Coutinho SR Minderico CS Matos MG Sardinha LB Teixeira PJ Exercise autonomous motivation predicts three-year weight loss in women Med Sci Sports Exerc 2010 10.1249/MSS.0b013e3181f3818f 20689448

[B40] Edmunds J Ntoumanis N Duda JL Adherence and well-being in overweight and obese patients referred to an exercise program on prescription scheme: a self-determination theory perspective Psychol Sport Exerc 2007 8 722 740 10.1016/j.psychsport.2006.07.006

[B41] Silva MN Markland D Minderico CS Vieira PN Castro MM Coutinho SR Santos TC Matos MG Sardinha LB Teixeira PJ A randomized controlled trial to evaluate self-determination theory for exercise adherence and weight control: rationale and intervention description BMC Public Health 2008 8 234 10.1186/1471-2458-8-234 18613959 PMC2483280

[B42] Lim BSC Wang JCK Perceived autonomy support, behavioural regulations in physical education and physical activity intention Psychol Sport Exerc 2009 10 52 60 10.1016/j.psychsport.2008.06.003

[B43] Deforche B Haerens L De Bourdeaudhuij I How to make overweight children exercise and follow the recommendations International Journal of Obesity in press 10.3109/17477166.2011.583660 21905814

[B44] Markland D Self-determination moderates the effects of perceived competence on intrinsic motivation in an exercise setting J Sport Exerc Psychol 1999 21 351 361

[B45] Hagger MS Chatzisarantis NLD Advances in self-determination theory research in sport and exercise Psychol Sport Exerc 2007 8 597 599 10.1016/j.psychsport.2007.06.003

[B46] Droomers M Schrijvers CTM Mackenbach JP Educational level and decreases in leisure time physical activity: predictors from the longitudinal GLOBE study J Epidemiol Community Health 2001 55 562 568 10.1136/jech.55.8.562 11449013 PMC1731951

[B47] Silva MN Vieira PN Coutinho SR Minderico CS Matos MG Sardinha LB Teixeira PJ Using self-determination theory to promote physical activity and weight control: a randomized controlled trial in women J Behav Med 2010 33 110 122 10.1007/s10865-009-9239-y 20012179

